# CDC Grand Rounds: Public Health Approaches to Reducing U.S. Infant Mortality

**Published:** 2013-08-09

**Authors:** Wanda Barfield, Denise D’Angelo, Rachel Moon, Michael Lu, Betty Wong, John Iskander

**Affiliations:** Div of Reproductive Health, National Center for Chronic Disease Prevention and Health Promotion, CDC; Academy of Pediatrics; Maternal and Child Health Bur, Health Resources and Svcs Admin; Office of the Associate Director for Science, CDC

Infant mortality is defined as the death of an infant before his or her first birthday. The infant mortality rate (IMR) measures this occurrence per 1,000 live births. In addition to being a key marker of maternal and child health, the IMR has been called the most sensitive indicator of overall societal health. In the United States, substantial progress has been made over the last 50 years in reducing the IMR; however, further reduction of preventable infant deaths remains a challenge. Based on preliminary data, the IMR in 2011 had declined to 6.05 overall, but that number obscures persistent racial and geographic disparities (Figure 1) (1). Non-Hispanic black infants continue to die at nearly twice the rate of non-Hispanic white infants. Additionally, preterm-related causes of death among black infants occur at a rate three times greater than that of white infants (2,3). Geographically, the majority of states in the top quartile for infant mortality are in the southern United States (Figure 2) (2).

Infant mortality is divided into two age periods: neonatal (birth–27 days) and postneonatal (28–364 days). Approximately two thirds of all infant deaths occur in the neonatal period, and one third occur in the postneonatal period. Infant deaths in the neonatal period are caused by complications arising from preterm births, birth defects, maternal health conditions, complications of labor and delivery, and lack of access to appropriate care at the time of delivery. Infant deaths in the postneonatal period are driven by sudden unexpected infant death (SUID) (including sudden infant death syndrome [SIDS]), injury, and infection. An increasing proportion of postneonatal infant deaths occur among infants who were born preterm but survived the neonatal period (4). Prevention of infant deaths should begin in the preconception period; opportunities are available to improve the health of mothers, and thus avoid preventable infant deaths.

## Infant Mortality in the United States

**FIGURE 3:**
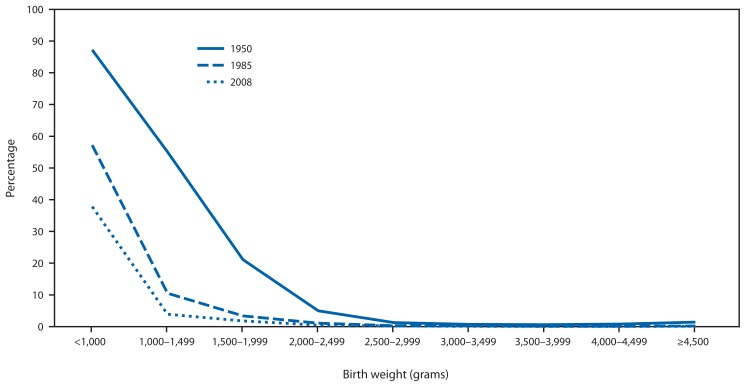
Birthweight-specific neonatal mortality — United States, 1950, 1985, and 2008

A major contributor to the decline in U.S. infant mortality is the decline in neonatal deaths associated with low birthweights (Figure 3). An infant born in 1950 with a birthweight <1,000 grams had only a 10%–15% chance of survival, whereas an infant born in 2008 with a birthweight <1,000 grams had a >60% chance of surviving the neonatal period.

Since 1950, medical technology has helped to reduce infant mortality, but the United States still has a relatively poor global standing. In 2010, the United States ranked 32nd among the 34 nations of the Organization for Economic Cooperation and Development in infant mortality, and the overall IMR was three times that of the countries with the lowest IMRs: Iceland (2.2 per 1,000), Finland (2.3), and Japan (2.3) (5). The main reason that the U.S. IMR remains higher than that of European nations is because the United States has a high percentage of preterm births (6). The United States ranks 130th out of 184 countries for preterm births; approximately 12% of U.S. births are preterm.*

Opportunities exist to reduce the mortality rate among infants born preterm by addressing key risk factors. Prenatal smoking contributes to low birthweight, preterm delivery, preterm-related death, and SIDS. Many very low birthweight infants in the United States are not born in hospitals that have level III neonatal intensive-care units, which have been shown to significantly reduce mortality (7). To reduce the IMR, the rates of preterm birth, including rates of late preterm birth (births between 34 and 36 weeks of gestation), need to be reduced (8).

## State and Local Efforts to Reduce Deaths in Infancy

The Pregnancy Risk Assessment Monitoring System (PRAMS) has been operating for the past 25 years.† In 1987, Congress appropriated funds for CDC to administer state-based programs of surveillance to collect data that would be helpful for reducing maternal and infant morbidity and mortality. Data collected would be used to direct the efforts of health programs. Forty states and New York City currently participate in PRAMS, and three additional states (California, Nevada, and Idaho) conduct PRAMS-like surveys. Information is reported to PRAMS by new mothers in response to a mailed questionnaire; if no response to the mailed questionnaire is received, the mother is contacted by telephone. Approximately 77,000 responses are received annually. In 2010, response rates ranged from 54% (Virginia) to 83% (Vermont). Key survey questions focus on topics such as breastfeeding, prenatal care, cigarette smoking during pregnancy, and infant sleep position.

Through the years, local and state efforts have been successful in identifying infant mortality risks through the use of PRAMS data. For example, white mothers and mothers aged <19 years have had the highest prevalence of smoking during pregnancy. Among PRAMS participants, West Virginia has had the largest percentage (>30%) of respondents who smoked cigarettes during the last 3 months of pregnancy, compared with New York City, which had the smallest percentage (2.3%). To help reduce smoking among pregnant women, West Virginia launched the “Tobacco Free Pregnancy Initiative” in 2009, with resulting increases in calls to tobacco quitlines by pregnant women and their families.§ In Michigan, PRAMS data revealed that black non-Hispanic mothers were 20% less likely than mothers of other races/ethnicities to place infants on their backs to sleep. In 2004, the Michigan governor’s office launched the “Infant Safe Sleep Campaign,” which included educational and policy components. Safe sleep messages were integrated into state services and programs. As one example, Michigan required child care centers to adhere to safe sleep recommendations as a condition for licensure.¶ Each of these state programs used PRAMS data to discover prevention opportunities and target interventions.

## Reducing SUID and SIDS

SUID comprises three main categories of death: accidental suffocation and strangulation in bed (ASSB), ill-defined deaths, and SIDS. Approximately 4,500 deaths per year are attributable to SUID. Of these deaths, approximately half result from SIDS, which is the leading cause of infant mortality in the postneonatal period. During 1995–2005, the proportion of SUID deaths attributed to SIDS declined, and the proportion of deaths attributed to ill-defined causes and ASSB increased.

Rates of SIDS and ASSB are highest among American Indians/Alaska Natives, followed by non-Hispanic blacks (9). Risk factors for SIDS include side or stomach sleeping position, bed sharing, soft bedding, and exposure to smoke, as well as prenatal drug or alcohol use by the mother. Protective actions against SIDS include room sharing without bed sharing, breastfeeding, pacifier use, and immunization. Based on epidemiologic studies, the American Academy of Pediatrics (AAP) published revised recommendations in October 2011, with level A recommendations considered the strongest recommendations (10). AAP recommends putting infants on their backs to sleep every time, using a firm sleeping surface, room sharing without bed sharing, keeping soft objects away from infants, regular prenatal care, breastfeeding, avoiding smoke exposure, and breastfeeding as much and for as long as a mother can. Additional level A recommendations include offering a pacifier at nap time and bedtime, avoiding overheating, and refraining from the use of home cardiorespiratory monitors as a strategy for reducing the risk for SIDS. AAP expanded the national campaign to include a major focus on the safe sleep environment and ways to reduce the risks for all sleep-related infant deaths.

## National Efforts

From 2007 through 2011, the U.S. IMR declined approximately 3% per year, from 6.75 to 6.05 per 1,000 live births, as a result of targeted efforts. If the trend continues, the U.S. IMR would be reduced to 4.5 per 1,000 live births by the year 2020. The *Healthy People 2020* target is to reduce the rate of infant death to 6.0 per 1,000 live births.** On June 14, 2012, the U.S. Department of Health and Human Services announced that it would work with state agencies to develop a national strategy for addressing infant mortality. The Secretary’s Advisory Committee on Infant Mortality was created as an independent body, administered by the Health Resources and Services Administration, to advise the Secretary on the programs created to reduce infant mortality. The committee provides the Secretary with advice on policies and resources required as well as advice on coordinating efforts with state agencies and programs. Strategies include 1) improving women’s health before pregnancy, 2) promoting quality and safety in prenatal care, 3) investing in prevention and health promotion, 4) promoting coordination among health services, 5) strengthening surveillance and research, and 6) promoting public/private and community collaboration.

The Collaborative Improvement and Innovation Network (CoIIN) to reduce infant mortality is a collaborative effort of states, the Health Resources and Services Administration, CDC, the Centers for Medicare & Medicaid Services, the Association of Maternal and Child Health Programs, the Association of State and Territorial Health Officials, the March of Dimes, and other agencies.†† CoIIN was initiated in 13 mostly southern states§§ in January 2012. Each state designs and shares their state’s plan to reduce infant mortality. The focus of CoIIN’s strategy teams include reducing elective deliveries at <39 weeks’ gestation, expanding interconception care in Medicaid, reducing SIDS/SUID, increasing smoking cessation among pregnant women, and expanding the regionalization of perinatal services to provide more appropriate levels of neonatal medical care for high-risk infants. High-risk infants need to be born in facilities where they can receive the best medical care, and decades of data demonstrate that high-risk infants born and cared for in level III neonatal intensive-care units have better outcomes. The goal of these programs and related collaborative efforts is to improve access to quality preconceptional, periconceptional, and prenatal health care across racial/ethnic and geographic divides, and to provide the best available care to mothers and infants.

## Figures and Tables

**FIGURE 1:**
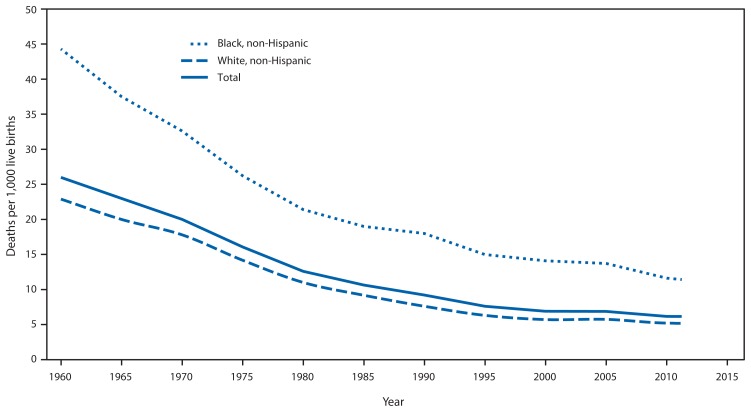
Infant mortality rates, by race/ethnicity and year — United States, 1960–2011

**FIGURE 2:**
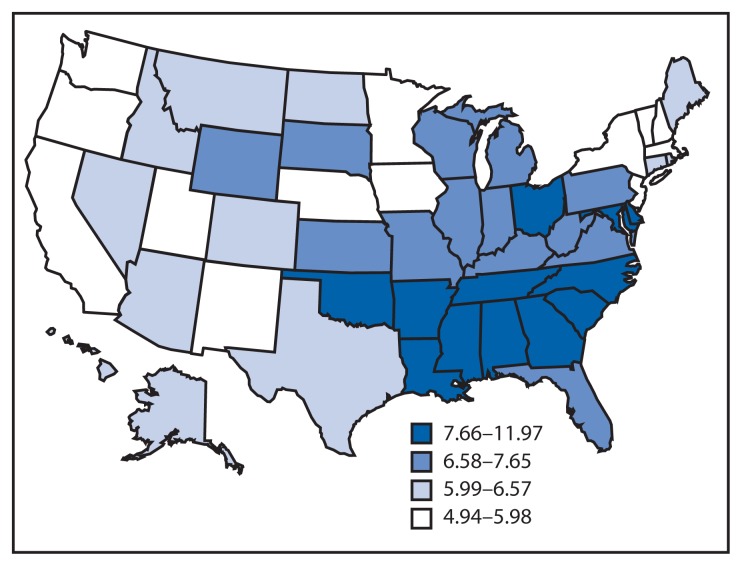
Infant mortality rates per 1,000 live births, by state — United States, 2004–2008

## Footnotes

* Additional information available at http://www.everywomaneverychild.org/borntoosoon.

† Additional information available at http://www.cdc.gov/prams.

§ Additional information available at http://c.ymcdn.com/sites/www.naquitline.org/resource/resmgr/2009_conference_materials/wvpostpartum.pdf.

¶ Additional information available at http://www.michigan.gov/dhs/0,4562,7-124-5453_7124_57836---,00.html.

** Additional information available at http://www.healthypeople.gov/2020/topicsobjectives2020/objectiveslist.aspx?topicid=26.

†† Additional information available at http://mchb.hrsa.gov/infantmortality/coiin/index.html.

§§ Alabama, Arkansas, Florida, Georgia, Kentucky, Louisiana, Mississippi, New Mexico, North Carolina, Oklahoma, South Carolina, Tennessee, and Texas.
